# Distinct T Cell Subset Profiles and T-Cell Receptor Signatures in Metabolically Unhealthy Obesity

**DOI:** 10.3390/ijms26073372

**Published:** 2025-04-04

**Authors:** Yoona Chung, Ji Yeon Chang, Shindy Soedono, Vivi Julietta, Esther Jin Joo, Soon Hyo Kwon, Sung Il Choi, Yong Jin Kim, Kae Won Cho

**Affiliations:** 1 Metabolic and Bariatric Surgery Center, Department of Surgery, H+ Yangji Hospital, Seoul 08779, Republic of Korea; 2 Soonchunhyang Institute of Medi-Bio Science (SIMS), Soonchunhyang University, Cheonan 31151, Republic of Koreashindysoedono@sch.ac.kr (S.S.);; 3 Department of Integrated Biomedical Science, Soonchunhyang University, Cheonan 31151, Republic of Korea; 4 Division of Nephrology, Department of Internal Medicine, Soonchunhyang University Seoul Hospital, Seoul 04401, Republic of Korea; 5 Department of Surgery, Kyung Hee University Hospital at Gangdong, Seoul 05278, Republic of Korea

**Keywords:** obesity, metabolically unhealthy obesity (MUO), metabolically healthy obesity (MHO), T cell, TCR repertoire, adipose tissue

## Abstract

Metabolically unhealthy obesity (MUO) is associated with increased inflammation and a higher risk of metabolic disorders compared to metabolically healthy obesity (MHO). T cell dysregulation in blood and adipose tissue may contribute to obesity-induced metabolic dysfunction, yet the characteristics of T cell subset profiles and T-cell receptor (TCR) repertoires in MHO and MUO remain unclear. We analyzed T cell subsets and TCR repertoires in peripheral blood and omental adipose tissue (oAT) from age- and BMI-matched MHO and MUO individuals using flow cytometry and high-throughput TCR sequencing. MUO individuals exhibited a higher proportion of memory CD4^+^ T cells in both compartments, with an increased frequency of central memory T cells. Circulating CD8^+^ T cells were increased in MUO, whereas CD8^+^ T cell subset composition remained unchanged in both blood and oAT. The TCR repertoire in oAT was significantly more restricted than in blood and showed greater skewing in MUO, with selective amplification of specific TRB V genes (TRBV12-4, TRBV18, TRBV7-9) and altered CDR3 length distributions. These findings suggest that distinct CD4^+^ T cell populations and specific TCR signatures may serve as potential biomarkers for metabolic dysfunction in obesity, providing insights into immune mechanisms underlying the transition from MHO to MUO.

## 1. Introduction

Severe obesity is considered an absolute public health priority worldwide according to the world health organization (WHO). According to the World Obesity Atlas 2023, approximately 24% of the global population, more than two billion people, is expected to have severe obesity (body mass index (BMI) ≥30 kg/m^2^) by 2035, which is an alarmingly increased rate compared to that of the reported 14% from 2020 [1]. The association of severe obesity with metabolic dysfunction, including insulin resistance, dyslipidemia, and hypertension is well known and severe obesity is therefore considered a risk factor for the development of type 2 diabetes mellitus (T2DM) and cardiovascular diseases [2]. However, accumulating evidence suggests that obesity does not always entail metabolic dysfunction or obesity-related cardio-metabolic complications [3]. There is a subgroup of individuals who retain a healthy metabolic profile despite severe obesity and have been designated as metabolically healthy obese (MHO). This term describes a specific subpopulation in which several or all the components of metabolic syndrome are absent. This favorable metabolic profile is most commonly characterized by a higher degree of insulin sensitivity, a lower prevalence of hypertension, and a favorable lipid profile in an individual with a BMI above a defined cut-off point based on ethnicity [3,4,5]. While MHO can be identified through different characteristics, a clear differential identification between MHO and metabolically unhealthy obesity (MUO) remains elusive.

Low-grade chronic inflammation originating from the adipose tissue is a well-established factor driving metabolic dysfunction and its variability among subgroups of obesity [6,7]. While adipose tissue macrophages have traditionally been considered the primary mediators to obesity-associated inflammation, emerging evidence highlights the critical role of adaptive immune system, particularly T cells, in sustaining this low-grade chronic inflammation and contributing to the development of T2DM [8,9,10,11]. Studies on morbidly obese individuals have shown an increased number of effector T cells and a higher proportion of circulating pro-inflammatory T cells, both of which positively correlate with obesity-related inflammation. Furthermore, individuals with concurrent obesity with T2DM exhibit notable alterations in T cell populations, including reduced frequencies of naïve T cells and an increased prevalence of effector memory T cells [9,12,13,14,15,16]. These changes collectively destabilize the homeostatic balance between cytotoxic effector and anti-inflammatory lymphocytes, thereby exacerbating systemic inflammation and impairing glucose tolerance. Supporting this, murine models have demonstrated that distinct adipose tissue T cell subsets contribute to obesity-associated inflammation and metabolic dysregulation [17,18,19]. These findings suggest that variations in T cell profiles, both in blood or adipose tissue, could serve as biomarkers and causal factors underlying the metabolic differences observed between MHO and MUO individuals. However, human studies investigating adipose tissue T cells in these subgroups remain limited, leaving a critical gap in understanding the mechanisms underlying obesity-related metabolic dysregulation.

Beyond T cell subset composition, the focus on identifying unique and specific T cells has grown significantly, given their crucial roles in the pathogenesis of various diseases. Advances in molecular biology, immunology, and bioinformatics have enabled deeper insights into T cell diversity through T-cell receptor (TCR) repertoire analysis in disease state. For instance, investigations into the blood TCR repertoire in type 1 diabetes mellitus (T1DM) have successfully identified distinct clonotypes and specific TCR signatures, highlighting their potential as pathogenic T cells and their causal relationships with particular antigens [12,20]. However, studies on the TCR repertoires in obesity remain limited, with most relying on peripheral blood [19,21]. This limitation emphasizes a critical gap in understanding, especially in light of studies demonstrating that tissue-resident TCR repertoires, such as those associated with tumors, offer higher specificity and sensitivity compared to their blood-derived counterparts [22]. In obese animal model, visceral adipose tissue (VAT) undergoes pronounced immunological transformations, including an accumulation of antigen-stimulated CD8^+^ T cells and exhausted CD4^+^ T cells, as well as significant alterations in the TCR repertoire unique to VAT but absent in blood or spleen [19,23,24,25]. These findings have repeatedly linked adipose tissue-specific TCR repertoires with metabolic dysfunctions such as insulin resistance, systemic inflammation, and cardiovascular events [19,26,27,28]. Consequently, there is an urgent need to expand research into TCR profiling within both peripheral blood and adipose tissues in individuals with severe obesity, with and without T2DM. Such studies will provide critical insights into the underlying mechanisms of metabolic dysfunction in MUO and may reveal novel biomarkers or therapeutic targets.

Given the crucial role of T cell profiles and TCR repertoires in obesity-associated inflammation and T2DM development, we hypothesized that MHO and MUO individuals exhibit distinct T cell compositions and TCR repertoires. This study aimed to comprehensively characterize the T cell composition and the clonal diversity of TCR repertoires in peripheral blood and adipose tissue from MHO and MUO individuals using advanced flow cytometry and high-throughput TCR sequencing.

## 2. Results

### 2.1. Demographic and Clinical Characteristics of Participants

The demographic and clinical characteristics of the cohort subjects are presented in Table 1. In both cohorts 1 and 2, the mean age and BMI of the two groups were comparable. Waist circumference and waist-to-hip ratio were also similar. As expected, the HbA1c levels and fasting glucose levels were significantly higher in the MUO group compared with that of the MHO group. Additionally, MUO group had significantly higher triglyceride levels and lower HDL-cholesterol levels than MHO group. There was no significant difference in blood pressure between the MHO group and the MUO group.

### 2.2. Alterations of T Cell Subset Composition in Peripheral Blood from MUO

**Figure 1:**
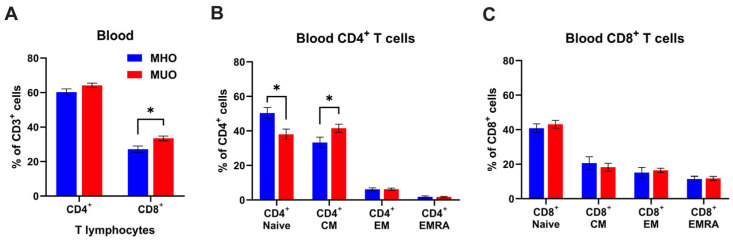
T cell profile in peripheral blood of MHO and MUO patients with type 2 diabetes. (**A**) Quantification of CD4^+^ and CD8^+^ cell frequencies among CD3^+^ T lymphocytes in blood; (**B**) quantification of CD4^+^ T cell subsets among CD4^+^ T cells; (**C**) quantification of CD8^+^ T cell subsets among CD8^+^ T cells. Data are presented as means ± standard error of the mean (SEM). MHO: metabolically healthy obesity (*n* = 10); MUO: metabolically unhealthy obesity (*n* = 18). T_EM_: T effector memory, T_CM_: T central memory, T_EMRA_: T effector memory revertant/re-expressing CD45-RA. Data are means ± SEM. * *p* < 0.05.

The strong association of obesity with inflammation is well established; however, the immunological characterization of MHO and MUO remains unclear. Since T lymphocytes are key players in adaptive immunity, we first examined whether circulating T lymphocyte levels would be different between MHO and MUO. Flow cytometry analysis demonstrated that circulating CD4^+^ and CD8^+^ T cells frequencies were ~3.9% and ~6.2%, higher in MUO individuals compared to MHO individuals (Figure 1A). A statistical difference in CD8^+^ T cell frequency was found between MHO and MUO (*p* < 0.05). A lower proportion of naïve CD4^+^ T cells, and a higher proportion of central memory T cell (T_CM_) was observed in MUO compared to MHO (Figure 1B). In contrast, CD8^+^ T cell subset composition was comparable between MUO and MHO (Figure 1C). These observations indicate that the shift from naïve T cells to effector/memory CD4^+^ T cells would be associated with MUO.

### 2.3. Alterations of CD4^+^ T Cell Subset Composition in Omental Adipose Tissue from MUO

**Figure 2:**
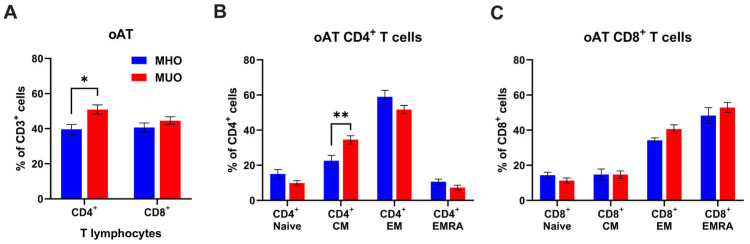
T cell profile in omental adipose tissue (oAT) of MHO and MUO patients with type 2 diabetes. (**A**) Quantification of CD4^+^ and CD8^+^ cell frequencies among CD3^+^ T lymphocytes in omental adipose tissue (oAT); (**B**) quantification of CD4^+^ T cell subsets among CD4^+^ T cells; (**C**) quantification of CD8^+^ T cell subsets among CD8^+^ T cells. Data are presented as means ± standard error of the mean (SEM). MHO: metabolically healthy obesity (*n* = 10); MUO: metabolically unhealthy obesity (*n* = 18). T_EM_: T effector memory, T_CM_: T central memory, T_EMRA_: T effector memory revertant/re-expressing CD45-RA. Data are means ± SEM. * *p* < 0.05; ** *p* < 0.01.

Given the distinct composition and phenotypes of T lymphocytes in tissues compared to circulating T cells, we proceeded to analyze the proportions of CD4^+^ and CD8^+^ T cells, along with their subset composition, in oAT from MHO and MUO. As shown in Figure 2, oAT CD4^+^ T cell frequency decreased (60% vs. 40%), but CD8^+^ T cell frequency increased (27% vs. 40%) in MHO, compared to the peripheral blood T cells from MHO. We also found that CD4^+^ T cells were significantly increased in oAT from MUO individuals, but there was no difference in the percentage of CD8^+^ T cells between MHO and MUO groups (Figure 2A). In the CD4^+^ T cell compartment, the frequencies of central memory CD4^+^ T cells were significantly higher in MUO individuals (Figure 2B). However, we did not observe any difference in CD8^+^ T cell subset composition between groups (Figure 2C). Taken together, our data demonstrate that MUO is associated with a shift toward central memory of CD4^+^ T cells in peripheral blood and oAT.

### 2.4. Decreased TCR Repertoire Diversity of Adipose CD4^+^ T Cells in MUO

**Figure 3:**
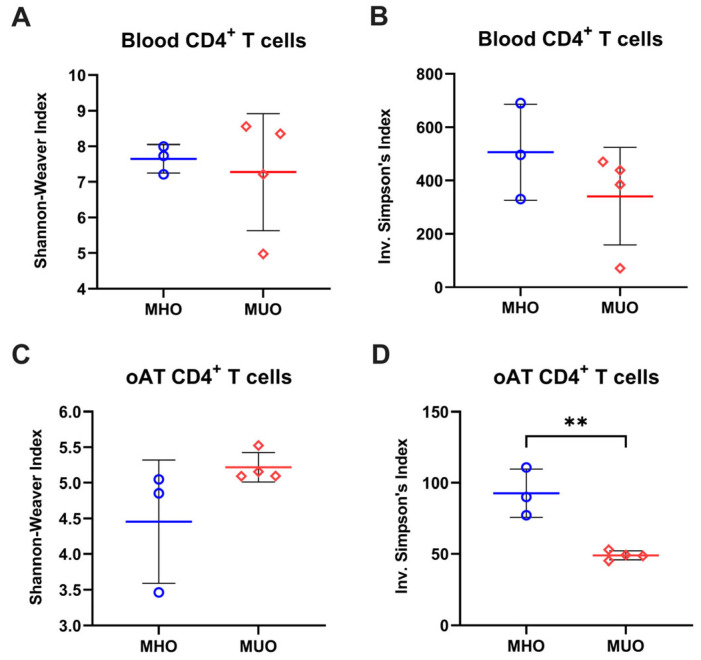
Diversity of T-cell immune repertoires in MHO and MUO patients with type 2 diabetes. (**A**) Shannon–Weaver index of CD4^+^ T cells in peripheral blood; (**B**) inverse Simpson’s index of CD4^+^ T cells in peripheral blood; (**C**) Shannon–Weaver index of CD4^+^ T cells in omental adipose tissue (oAT); (**D**) Simpson’s index of CD4^+^ T cells in oAT. Data are presented as means ± standard error of the mean (SEM). MHO: metabolically healthy obesity (*n* = 3); MUO: metabolically unhealthy obesity (*n* = 4). ** *p* < 0.01.

T cells are distinguishable by their unique TCR sequence. Based on the involvement of T_CM_ with MUO, we next analyzed the TCR repertoire of blood and oAT T cells to assess whether relative dominance of (auto)antigen-experience could be attributed to the accumulation of central memory T cell in MUO. Analysis using the Shannon index and inverse Simpson’s index revealed that both the richness and evenness of TCR clonotypes in blood T cells were reduced in oAT T cells, suggesting a more restricted TCR repertoire in AT compared to peripheral blood (Figure 3 and Figure S1). The Shannon index exhibited comparable values between MHO and MUO, while the inverse Simpson’s index showed a decreasing trend, indicating alterations in the blood TCR repertoire of MUO individuals (Figure 3A,B). In CD4^+^ T cells from oAT, the Shannon index appeared higher in the MUO group than in MHO groups, though variability was observed (Figure 3C). Conversely, the inverse Simpson’s index was significantly lower in the adipose TCR repertoire of MUO compared to MHO (Figure 3D). These findings suggest that adipose tissue from MUO exhibits a skewed TCR repertoire, indicating clonal expansion of T cells.

### 2.5. Characterization of Peripheral Blood and oAT TCR Repertoires of MUO Subjects

**Figure 4:**
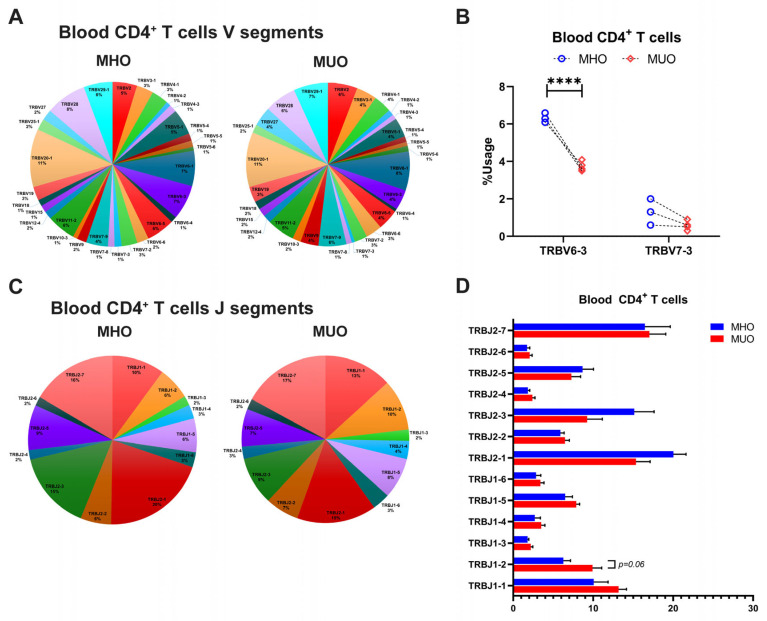
T-cell receptor beta (TRB) gene usage in peripheral blood of MHO and MUO patients with type 2 diabetes. (**A**) Venn diagram of TRB V segment gene usage in blood CD4^+^ T cells from MHO and MUO subjects; (**B**) the usage rate of TRB V genes between the MHO and the MUO groups in blood CD4^+^ T cells; (**C**) Venn diagram of TRB J segment gene usage in blood CD4^+^ T cells from MHO and MUO subjects; (**D**) the usage rate of TRB J genes between the MHO and the MUO groups in blood CD4^+^ T cells. Data are presented as means ± standard error of the mean (SEM). MHO: metabolically healthy obesity (*n* = 3); MUO: metabolically unhealthy obesity (*n* = 4). **** *p* < 0.0001.

The diversity of TCR repertoire is primarily determined by CDR3, which arises from somatic recombination of the variable (V), diversity (D), and joining (J) genes, along with nucleotide addition and deletion at the V–D and D–J junctions [29,30]. To explore the differences of TCR repertoire between MHO and MUO, we evaluated TRB chain gene usage, including TRBV, TRBJ, and TRB V-J pairs, across all subjects (Figures S2–S4). The analysis revealed that certain T cell TRBV genes, such as TRBV20–1, TRBV28, and TRBV6-1, were prevalently utilized in blood CD4^+^ T cells among all participants (Figure 4A). Notably, TRBV6-3 gene usages varied significantly between MHO and MUO subjects (Figure 4B), while several J segments (TRB J2-7, TRB J2-2, TRB J-1, and TRB J1-1) showed higher usage overall but without significant difference between groups (Figure 4C,D). Furthermore, specific V-J segment pairs, including V24-1-J1-2, V5-6-J2-5, V20-1-J1-1, V2-J1-5, V6-4-J2-7, and V2-J2-7, exhibited significantly increased usage in peripheral CD4^+^ T cells from MUO (Table 2).

**Figure 5:**
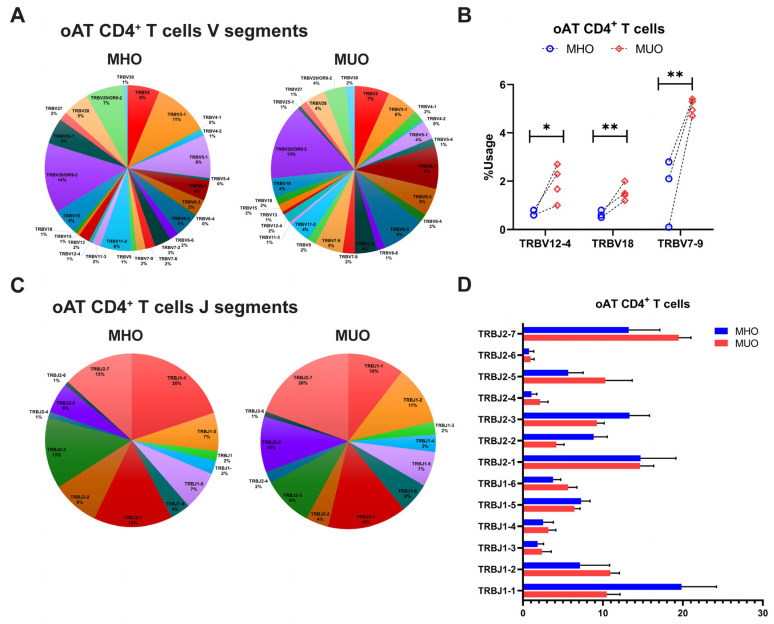
T-cell receptor beta (TRB) gene usage in omental adipose tissue (oAT) of MHO and MUO patients with type 2 diabetes. (**A**) Venn diagram of TRB V segment gene usage in oAT CD4^+^ T cells from MHO and MUO subjects; (**B**) the usage rate of TRB V genes between the MHO and the MUO groups in oAT CD4^+^ T cells; (**C**) Venn diagram of TRB J segment gene usage in oAT CD4^+^ T cells from MHO and MUO subjects; (**D**) the usage rate of TRB J genes between the MHO and the MUO groups in oAT CD4^+^ T cells. Data are presented as means ± standard error of the mean (SEM). MHO: metabolically healthy obesity (*n* = 3); MUO: metabolically unhealthy obesity (*n* = 4). * *p* < 0.05; ** *p* < 0.01.

Consistent with the findings on TCR repertoire diversity, the overall frequency of V gene usage was diminished in adipose CD4^+^ T cells compared to peripheral counterparts, with only select V genes being used at higher frequencies (Figure 5A). In the AT of MUO, TCR sequencing identified increased usage of TRBV genes such as TRBV7-9, TRBV18, and TRBV 12-4, although TRBV20-1 gene usage remined relatively higher across all subjects (Figure 5A,B). Comparisons of J segment usage showed no significant differences between MHO and MUO (Figure 5C,D). However, the MUO group displayed significantly higher frequencies of several TRB V-J gene pairs, including TRB V9-J2-2 and V10-3-J1-1, relative to the MHO group (Table 3).

Collectively, these results indicate that distinct utilization patterns of TRB genes, particularly V gene and V-J segment pairs, may contribute to the restricted TCR diversity observed in individuals with MUO.

### 2.6. Different CDR3 Amino Acid Sequences in MUO

**Figure 6:**
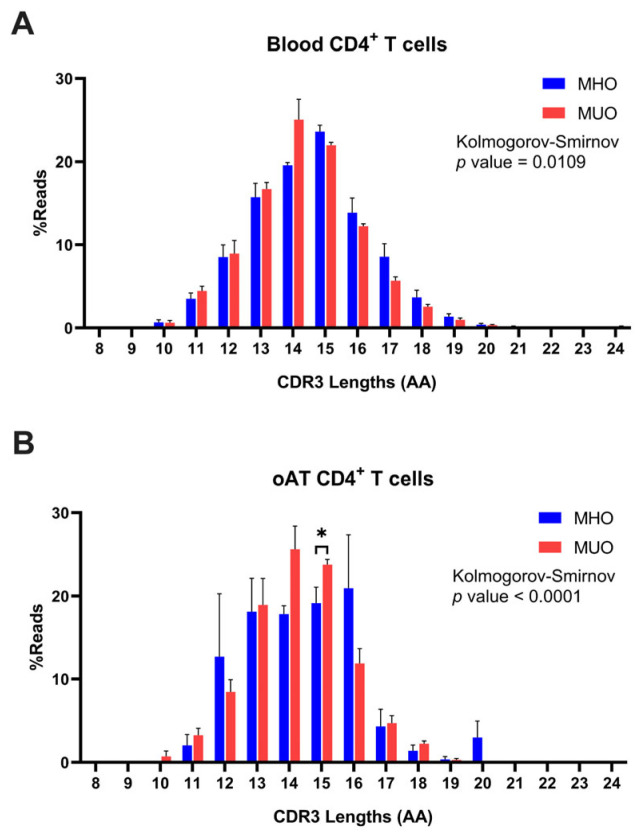
Distribution of T-cell immune repertoires based on CDR3 amino acid (AA) length in MHO and MUO patients with type 2 diabetes. (**A**) Percentage distribution of CDR3 amino acid lengths (AA) in peripheral blood CD4^+^ T cells; (**B**) percentage distribution of CDR3 amino acid lengths (AA) in omental adipose tissue (oAT) CD4^+^ T cells. Data are means ± SEM. * *p* < 0.05.

Further analysis was conducted on TCR CDR3 clonotypes derived from both peripheral blood and AT of MHO and MUO individuals. The range of CDR3 length was observed to span from 5 to 25 amino acids (AAs), with a normal distribution peaking at a mode of 15 AAs (Figure 6A). Although CDR3 length distributions were comparable among subjects from both groups, the peripheral T cells of MUO exhibited a trend toward shorter CDR3 lengths compared to those from MHO individuals (MHO vs. MUO, 15 vs. 14). To further assess this observation, we conducted an additional analysis using the Kolmogorov–Smirnov test, a non-parametric method for comparing distributions. The analysis indicated a statistically significant difference between the groups, although the overall distribution graphs do not show a pronounced shift.

In line with the findings from peripheral blood T cells, the analysis of AT T cells revealed a median CDR3 length of 15 AAs in MHO subjects and 14 AAs in MUO subjects (Figure 6B). This consistency highlights the trend across both tissue types, suggesting alterations in the clonal structures of T cells in MUO individuals. Intriguingly, differential distribution of CDR3 length in AT T cells between two groups was observed, which may indicate unique clonal characteristics associated with adipose tissue in MUO.

## 3. Discussion

The association between obesity and adaptive immune system dysregulation is well established. While alterations in T cell subsets in obese murine models and human obesity have been extensively studied [14,19,31], the nuanced distinctions among obesity subtypes remain unclear. In this study, we characterized T cell profiles and TCR repertoires in both peripheral blood and oAT of age- and BMI-matched MHO and MUO individuals. Our findings revealed a significant shift toward a higher proportion of memory CD4^+^ T cells in MUO, alongside a more restricted and skewed TCR repertoire in oAT compared to MHO. These results highlight the pivotal role of T lymphocytes and adaptive immunity not only in obesity, but also in the progression toward metabolic dysfunction in obese individuals.

Peripheral blood T cell composition in MUO showed a marked reduction in naïve CD4^+^ T cells, with a corresponding increase in effector/memory subsets, aligning with prior rodent studies [19,32,33]. Notably, T_CM_ CD4^+^ T cells were significantly elevated in MUO, while naïve CD4^+^ T cells were decreased. This contrasts with previous studies in morbid obesity that documented increases in both subsets [34]. The discrepancies observed may stem from differences in study populations, particularly variations in BMI and age. Unlike CD4^+^ T cells, total CD8^+^ T cells in peripheral blood exhibited a significant increase in MUO, even though the proportions of distinct CD8^+^ T cell subsets (central memory and effector memory) remained relatively stable (Figure 1). Prior studies have reported enhanced CD8^+^ T cell differentiation in individuals with metabolic syndrome (MetS) [35], which may be influenced by aging, given its association with increased effector memory CD8^+^ T cell [36]. In our study, although the relative proportions of CD8^+^ T cell subsets did not show significant differences, the overall absolute number of CD8^+^ T cells was elevated in MUO subjects. This finding is consistent with previous reports linking metabolic dysregulation to CD8^+^ T cell expansion [35]. The absence of marked subset differentiation in our cohort may be related to the relatively young mean age (~30 years), indicating metabolic dysregulation can promote an increase in total CD8^+^ T cells independent of aging effects.

A logical consequence of these observed alterations in T cell subsets is the potential impact on cytokine production, which plays a critical role in driving the chronic inflammatory state associated with metabolic dysfunction. For instance, dysregulated T cell subsets have been shown to produce increased levels of pro-inflammatory cytokines such as TNF-α, IFN-γ, and IL-17 [8,37,38], while reductions in regulatory T cell populations correlate with lower anti-inflammatory cytokine levels such as IL-10 [39]. Although our study primarily focused on the phenotypic characterization of T cells, these findings suggest that the observed shifts in T cell composition in MUO individuals may contribute to an altered cytokine milieu that exacerbates metabolic dysfunction. Future studies incorporating direct measurements of cytokine production will be essential to confirm these mechanistic links.

Despite extensive studies in murine models, data on human T cell subsets in oAT remain limited, particularly in the context of metabolic dysfunction. We found that oAT was significantly enriched in CD4^+^ and CD8^+^ T_EM_ and T_EMRA_ cells compared to blood, with a relative depletion of naïve T cells (Figure 2). Notably, CD4^+^ T_CM_ were significantly elevated in MUO, supporting their involvement in obesity-associated inflammation, insulin resistance, and type 2 diabetes. These findings extend prior studies on subcutaneous adipose tissue (SAT), where T_EM_ cells have been linked to insulin resistance in obesity [34,36], reinforcing the role of adipose tissue-resident T cells in metabolic dysfunction. The high prevalence of these subsets in oAT indicates localized antigenic stimulation, emphasizing their contribution to systemic inflammation.

TCR repertoire analysis further elucidated the immunological landscape of adipose tissue in obesity. Consistent with murine models [31] and prior studies in individuals with T2DM [14,19], we observed a significantly reduced TCR repertoire diversity in oAT compared to peripheral blood, with greater skewing in MUO. This suggests that adipose tissue-resident T cells may undergo antigen-driven clonal expansion. Additionally, we identified distinct TCR features between MHO and MUO, including differential TRB V gene usage, V-J gene pair frequencies, and CDR3 length distributions. Notably, specific TCR fragments (e.g., TRB V12–4, TRB V18, and TRB V7-9) were selectively amplified in oAT from MUO individuals, with TRBV12–4 and TRBV12–3 previously implicated in antigen-specific responses in type 1 diabetes [40,41]. Similar to the AT, specific V-J gene pairs, such as V20-1-J1-1 and V5-1-J2-4, were highly enriched in peripheral CD4^+^ T cells from MUO individuals. These observations align with reports in diabetic Pima Indians, where TRBV20–1, TRBV5–1, TRBJ2–1, and TRBJ2–4 were found to be frequently utilized [42,43]. Collectively, these findings suggest that distinct T cell population expressing specific TCRs may serve as potential biomarkers for diabetes risk and obesity-associated metabolic dysfunction. While our data indicate a clear alteration in TCR repertoires, establishing the precise antigen specificity and mechanistic contributions of these TCR changes will require further investigation. With rapid advances in sequencing technologies and computational analyses, future studies will be better positioned to address these questions and clarify the link between antigen-driven T cell responses and metabolic dysfunction.

Several limitations should be considered. First, our study was conducted at a single center with a relatively small sample size, which may limit the statistical power to detect certain differences. However, these samples were carefully selected using strict criteria (e.g., age, BMI, and other relevant factors) to minimize confounding variables, thereby enhancing the interpretability of our findings despite the limited numbers. Second, the stability of MHO in our cohort remains unknown, and longitudinal studies are needed to assess the transition from MHO to MUO. Third, functional characterization of identified TCR clonotypes was not performed, preventing direct assessment of their role in T2DM pathogenesis. Finally, we did not evaluate potential cross-reactivity of T cells due to autoimmune mechanisms or bystander effects [44,45], warranting further investigation.

In conclusion, our findings provide novel insights into the role of T cells in obesity-associated metabolic dysfunction, extending previous murine studies to human data. Specifically, the enrichment of memory CD4^+^ T cells in both blood and adipose tissue of MUO individuals underscores their immunological contribution to metabolic disease. Furthermore, TCR repertoire analysis highlights distinct adaptive immune signatures in MUO, laying the groundwork for personalized diagnostic and therapeutic approaches targeting T cell-mediated pathways in metabolic disorders.

## 4. Materials and Methods

### 4.1. Study Design and Participants

Twenty-eight patients with severe obesity undergoing bariatric surgery were recruited and grouped into the MHO and MUO groups. MHO was determined based on the suggested definition criteria with a diagnosis of obesity (BMI ≥ 30 kg/m^2^): waist circumference >90 cm; waist-to-hip ratio >0.9; fasted serum triglycerides ≤1.7 mmol/L (≤150 mg/dL); HDL-cholesterol serum concentration >1.0 (>40 mg/dL) in men or >1.3 mmol/l (>50 mg/dL) in women; systolic blood pressure (SBP) ≤135 mmHg; diastolic blood pressure ≤85 mmHg; fasting blood glucose ≤6.1 mmol/L (≤100 mg/dL); no medication for dyslipidemia, diabetes, or hypertension; and no cardiovascular disease manifestation [5]. For the MUO groups, obese patients with T2DM were used for analysis. Peripheral blood-derived and omental adipose tissue (oAT) T lymphocyte profiles were examined (cohort 1). TCR sequencing of peripheral blood-derived and oAT CD4^+^ T cell was performed in another age- and BMI-matched MHO and MUO subjects (cohort 2). The protocol for this research was reviewed and approved by the Institutional Review Board of Soonchunhyang University Seoul Hospital (IRB # 2015-11-020) and conformed to the provisions of the Declaration of Helsinki. A written informed consent was obtained from each participant before undergoing metabolic bariatric surgery.

### 4.2. Acquisition and Isolation of Adipose Tissue and Blood T Cells

Venous blood samples and oAT were collected before and during surgery, respectively. The cells from stroma-vascular fraction (SVF) were isolated from the adipose tissue as previously described [46]. Briefly, the adipose tissue was mechanically minced using a gentleMACS™ Dissociator (Miltenyi Biotec, Germany), enzymatically digested (complete medium (RPMI1640, 10% FBS, 100 U/mL penicillin, 100 ug/mL streptomycin, 2 mM L-glutamine) with 1 mg/mL collagenase 1 and 0.1 mg/mL DNase for 1 h at 37 °C in a mechanical shaker), and centrifuged to attain the stromal vascular cells. For the peripheral blood, the mononuclear cells were isolated by density gradient centrifugation on Ficoll-Paque™ Plus (GE Healthcare, Chicago, IL, USA). CD3^+^ T cells and CD4^+^ T cells from AT-SVFs and blood were isolated using immunoselection/depletion protocol as previously described [18,47]. CD3^+^ T cells from AT-SVF and blood were selected on the CD14 negative cell fraction and followed by CD3^+^ positive selection (STEMCELL Technologies, Vancouver, BC, Canada). CD4^+^ T cells were enriched from all samples using magnetic negative selection for CD3^+^ T cells and followed by positive selection for CD4^+^ T cells (Miltenyi Biotec, Germany). Cell numbers were counted using trypan blue staining and the purity of cells was confirmed by flow cytometry. Isolated blood and adipose CD4^+^ T cells were immediately processed to RNA isolation or stored at −80 °C.

### 4.3. Flow Cytometry Analysis

For flow cytometry of T cell subsets, cells were stained with fluorescent-labeled anti-human antibodies (CD3-APC-H7, CD4-PerCp-Cy5.5, CD8-PE, CCR7-Pe-CF494, CD45RA-APC, and CD62L-alexaFluor700), along with appropriate isotype-matched control antibodies. All fluorochrome-conjugated antibodies and reagents were purchased from either BD Biosciences or Biolegend. Analyses were performed using BD FACSAria II insturment (BD Biosciences, San Jose, CA, USA) and Flowjo software (Tree Star, Portland, OR, USA). The T cell subsets populations in our study are defined as naïve T cells (CD45RA^+^CCR7^+^CD62L^+^), T effector memory (T_EM,_ CD45RA^−^CCR7^−^CD62L^−^), T central memory (T_CM_, CD45RA^−^CCR7^+^CD62L^+^), and T effector memory revertant/re-expressing CD45-RA (T_EMRA_, CD45RA^+^CCR7^−^CD62L^−^).

### 4.4. RNA Isolation and High-Throughput Sequencing of TCRs

RNA was extracted from blood and adipose CD4^+^ T cells using TRIzol. The RNA quantity and purity were measured using an Agilent RNA 6000 Nano kit and 2100 Bioanalyzer (Agilent technologies, Santa Clara, CA, USA). The TCR β chain of CDR3 RNA sequencing libraries were constructed by multiplex PCR amplification. The sequencing process covered CDR3, which is rearranged by 48 V and 13 J gene segments of the TCR β chain. The qualities of the libraries were assessed according to the following criteria: the library concentration ≥2 ng/μL, the main fragment size at approximately 300 bp, and no primer dimers (<100 bp) [48]. The libraries were finally sequenced using an Illumina Miseq paired-end platform (Repertoire Genesis Inc, Osaka, Japan).

### 4.5. Sequencing Data Preprocessing

The raw sequencing reads were demultiplexed based on the sequences of index primers corresponding to different samples. All paired-end reads were classified according to these index sequences. The sequence assignment was performed by identifying sequences with the highest identity in a reference dataset from the International ImMunoGeneTics information system^®^ (IMGT) database. Data processing, assignment, and aggregation were automatically handled using the Repertoire Genesis (RG) software developed by Repertoire Genesis Inc. Reads were compared against the IMGT database (www.imgt.org) which generated each TCRB read V and J gene usage and CDR3B length.

The diversity of the TCR was calculated using the Shannon–Weaver index and Simpson’s diversity index [49], which considered the influence of the number of unique receptors (richness) and their relative abundance (evenness).

### 4.6. Statistical Analysis

Continuous variables are presented as the mean ± standard error of the mean (SEM), and statistical calculations were performed using GraphPad Prism v10 (GraphPad Software, La Jolla, CA, USA). An unpaired Student’s *t*-test, Wilcoxon–Mann–Whitney test, or multiple *t*-tests with Bonferroni–Dunn correction were used to evaluate differences between groups. A significant difference was defined by *p* < 0.05. The significance of CDR3 distribution between MHO and MUO was analyzed using the Kolmogorov–Smirnov test.

## Figures and Tables

**Table 1:** Characteristics of the subjects for immune repertoire analysis.

Cohort 1	MHO ^a^ (*n* = 10)	MUO ^b^ (*n* = 18)	*p* Value
Gender (F/M, *n*)	2/8	5/13	
Age (mean, years)	33.1	30.7	0.462
BMI (mean, kg/m^2^)	39.3	39.2	0.985
Waist circumference (mean, cm)	122.125	118.733	0.617
Waist-to-hip ratio (mean, ratio)	0.953	0.963	0.706
HbA1c (mean, %)	5.3	6.9	0.025
Fasting plasma glucose (mean, mg/dL)	112	137.6	0.030
Systolic BP (mean, mmHg)	127	131	0.562
Diastolic BP (mean, mmHg)	74.6	77.3	0.492
Triglyceride (mean, mg/dL)	105.5	201.7	<0.001
HDL-cholesterol (mean, mg/dL)	52	39.6	0.005
Cohort 2 for TCR sequencing	MHO ^a^ (*n* = 3)	MUO ^b^ (*n* = 4)	*p* value
Gender (F/M, n)	2/1	4/0	
Age (mean, years)	33.7	32.7	0.912
BMI (mean, kg/m^2^)	35.9	35.0	0.741
Waist circumference (mean, cm)	118	111.5	0.619
Waist-to-hip ratio (mean, ratio)	0.965	1.018	0.159
HbA1c (mean, %)	5.2	10.1	<0.001
Fasting plasma glucose (mean, mg/dL)	92.3	161.5	0.030
Systolic BP (mean, mmHg)	133	127	0.696
Diastolic BP (mean, mmHg)	76.6	78	0.904
Triglyceride (mean, mg/dL)	129.5	203.0	0.183
HDL-cholesterol (mean, mg/dL)	57.3	39.0	0.048

^a^ MHO: metabolically healthy obesity, ^b^ MUO: metabolically unhealthy obesity.

**Table 2:** Differentially used TRB V-J pairs of blood CD4^+^ T cells.

TRB V	TRB J	*p* Value
TRB V24-1	TRB J1-2	0.018
TRB V5-6	TRB J2-5	0.019
TRB V20-1	TRB J1-1	0.022
TRB V2	TRB J1-5	0.039
TRB V6-4	TRB J2-7	0.044
TRB V2	TRB J2-7	0.048
TRB V7-9	TRB J1-5	0.052
TRB V6-1	TRB J2-5	0.065
TRB V5-1	TRB J2-4	0.071
TRB V12-3	TRB J2-5	0.071
TRB V10-1	TRB J2-7	0.075
TRB V7-6	TRB J1-5	0.075
TRB V14	TRB J2-5	0.091
TRB V13	TRB J1-6	0.092

**Table 3:** Differentially used TRB V-J pairs of adipose CD4^+^ T cells.

TRB V	TRB J	*p* Value
TRB V9	TRB J2-2	0.008
TRB V10-3	TRB J1-1	0.014
TRB V12-4	TRB J1-2	0.029
TRB V13	TRB J2-2	0.030
TRB V6-3	TRB J1-5	0.031
TRB V6-6	TRB J2-3	0.051
TRB V12-4	TRB J2-1	0.057
TRB V6-5	TRB J1-6	0.091
TRB V10-3	TRB J1-4	0.096

## Data Availability

Dataset available on request from the authors.

## Supplementary Materials

The following supporting information can be downloaded at: https://www.mdpi.com/article/10.3390/ijms26073372/s1.

## Abbreviations

The following abbreviations are used in this manuscript:

MUO	Metabolically unhealthy obesity
MHO	Metabolically healthy obesity
TCR	T-cell receptor
OAT	Omental adipose tissue
WHO	World Health Organization
BMI	Body mass index
T2DM	Type 2 diabetes mellitus
T1DM	Type 1 diabetes mellitus
VAT	Visceral adipose tissue
AT	Adipose tissue
V	Variable gene
D	Diversity gene
J	Joining gene
AAs	Amino acids
TCM	Central memory
MetS	Metabolic syndrome
SAT	Subcutaneous adipose tissue
SBP	Systolic blood pressure
HDL	High-density lipoprotein
SVF	Stroma-vascular fraction
T_EM_	T effector memory
T_CM_	T central memory
T_EMRA_	T effector memory revertant/re-expressing CD45-RA
IMGT	ImMunoGeneTics information system^®^
RG	Repertoire genesis
