# Supplementary material for: Distinct T Cell Subset Profiles and T-Cell Receptor Signatures in Metabolically Unhealthy Obesity
Source: Int J Mol Sci. 2025 Apr 4;26(7):3372. doi: 10.3390/ijms26073372 (PMC11989847; doi:10.3390/ijms26073372)
Supplement: Supplementary file 1 [file ijms-26-03372-s001.zip › ijms-3511520-supplementary.pdf]

SUPPLEMENTARY FIGURE

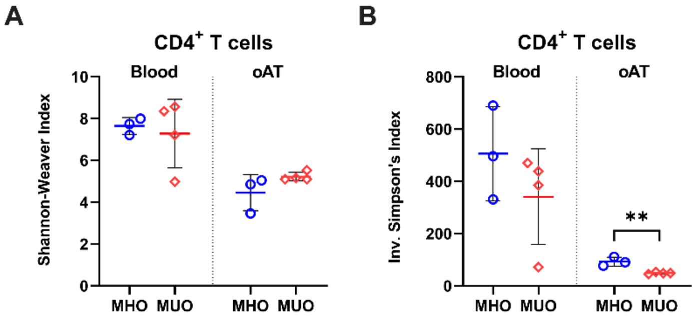

Figure S1. The diversity of T-cell immune repertoires. (a) Shannon-Weaver index from blood and omental adipose tissue (oAT) $CD4^+$ T cells **(b)** Inverse Simpson's index from blood and oAT $CD4^+$ T cells. Data are presented as means ± standard error of the mean (SEM). MHO: metabolically healthy obesity (*n*=3), MUO: metabolically unhealthy obesity (*n*=4).

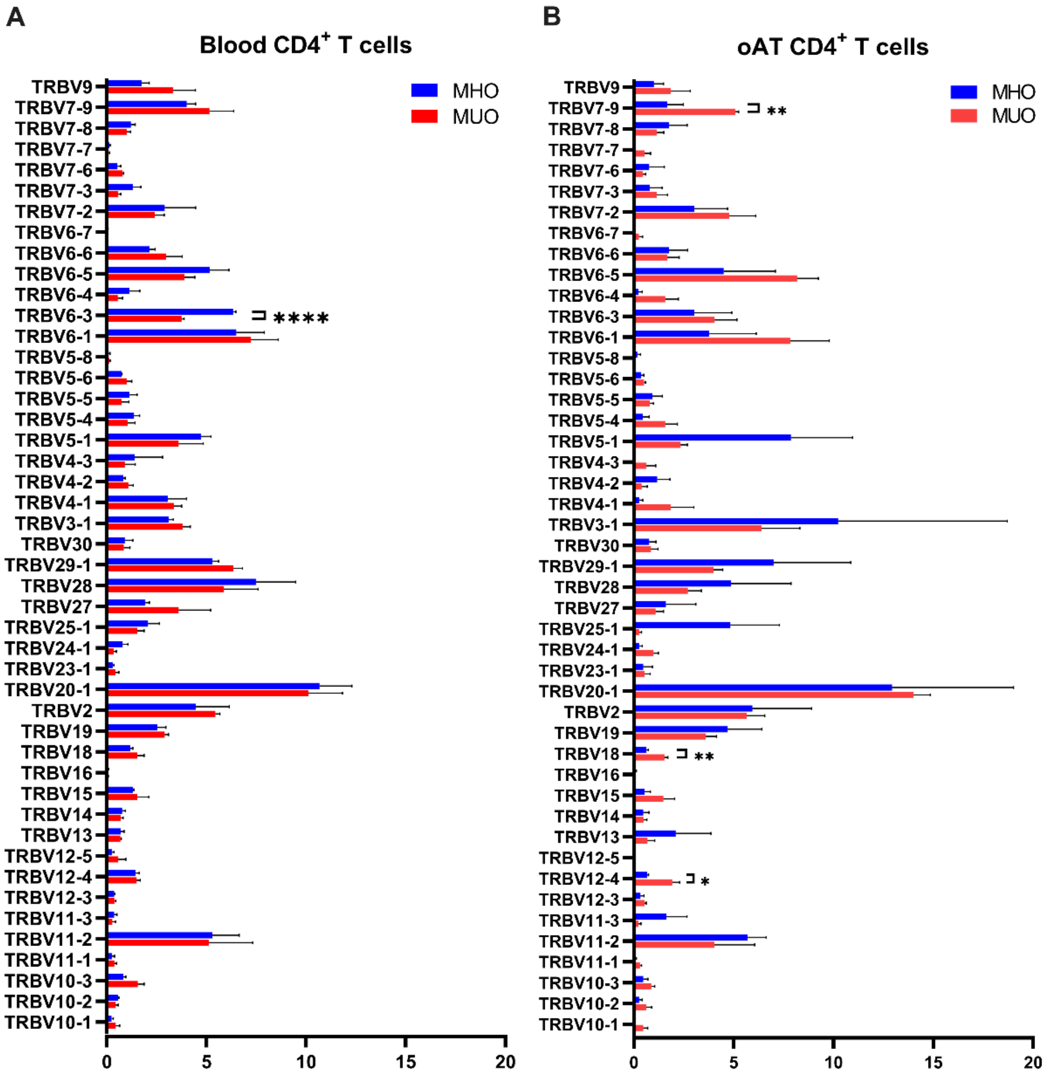

Figure S2. Comparison of TRB V genes usage in (a) peripheral blood and (b) omental adipose tissue (oAT) CD4^{+} T cells from MHO and MUO subjects. Data are presented as means ± standard error of the mean (SEM). MHO: metabolically healthy obesity (*n*=3), MUO: metabolically unhealthy obesity (*n*=4).

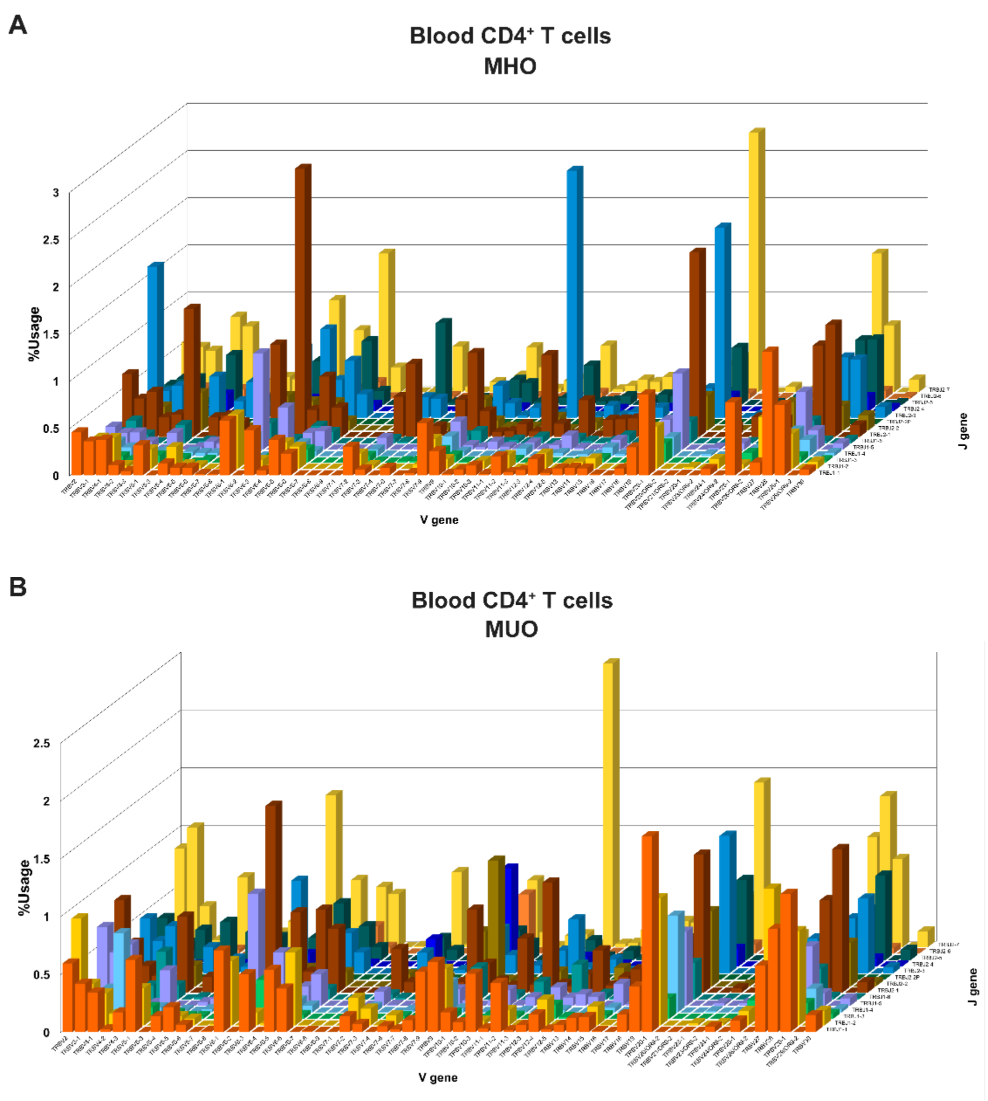

Figure S3. The average frequencies of TRB V-J pair usages in peripheral blood of MHO and MUO patients with type 2 diabetes. MHO: metabolically healthy obesity, MUO: metabolically unhealthy obesity.

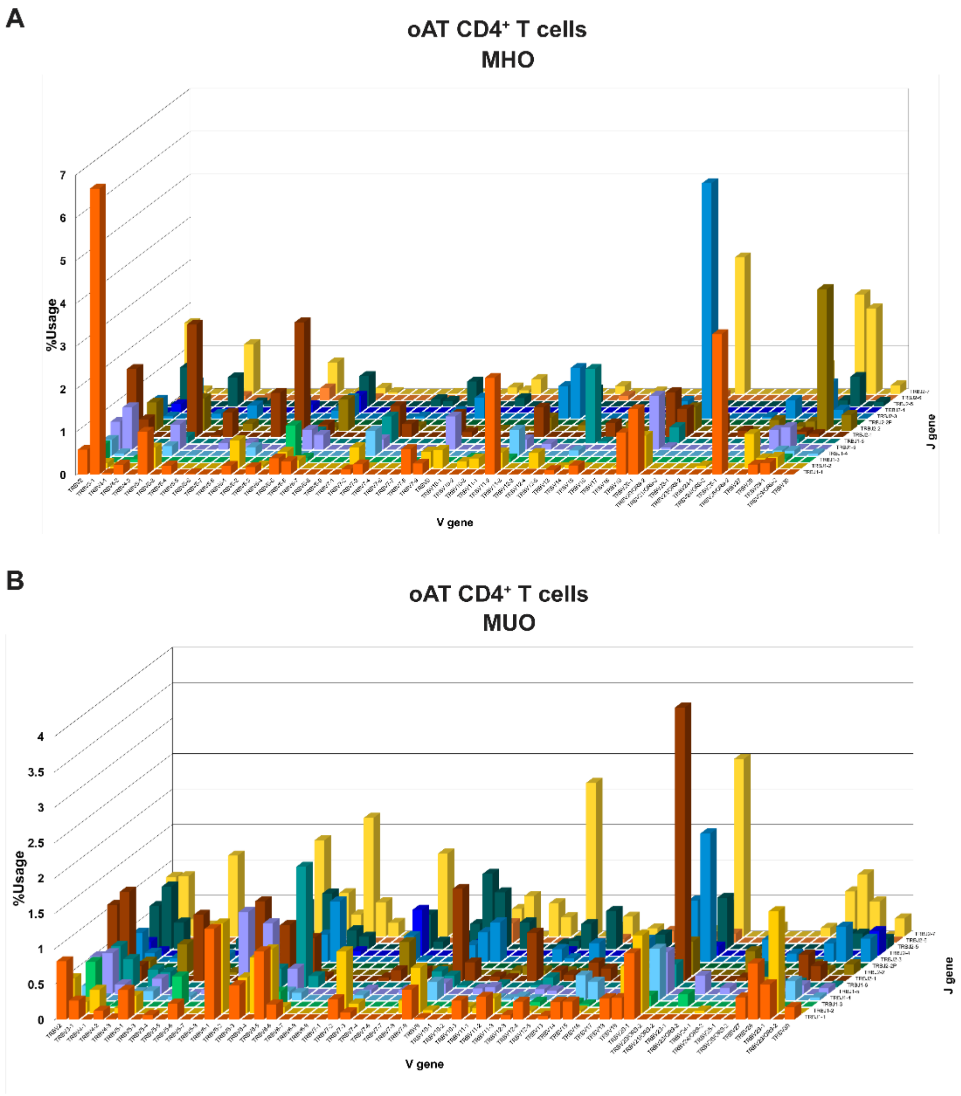

Figure S4. The average frequencies of TRB V-J pair usages in omental adipose tissue (oAT) of MHO and MUO patients with type 2 diabetes. MHO: metabolically healthy obesity, MUO: metabolically unhealthy obesity.
